# Deep learning algorithm reveals two prognostic subtypes in patients with gliomas

**DOI:** 10.1186/s12859-022-04970-x

**Published:** 2022-10-11

**Authors:** Jing Tian, Mingzhen Zhu, Zijing Ren, Qiang Zhao, Puqing Wang, Colin K. He, Min Zhang, Xiaochun Peng, Beilei Wu, Rujia Feng, Minglong Fu

**Affiliations:** 1 Hubei Clinical Research Center of Parkinson’s Disease, Xiangyang Key Laboratory of Movement Disorders, Xiangyang No.1 People’s Hospital, Hubei University of Medicine, Xiangyang, 441000 Hubei People’s Republic of China; 2 Data Science and Statistics, Stego Tech LLC, 422 Lynrose CT, King of Prussia, PA 19406 USA

**Keywords:** Autoencoder-based approach, Support vector machine, Survival-sensitive subtypes, Multi-omics data, Glutathione metabolism pathway

## Abstract

**Background:**

Gliomas are highly complex and heterogeneous tumors, rendering prognosis prediction challenging. The advent of deep learning algorithms and the accessibility of multi-omic data represent a new approach for the identification of survival-sensitive subtypes. Herein, an autoencoder-based approach was used to identify two survival-sensitive subtypes using RNA sequencing (RNA-seq) and DNA methylation (DNAm) data from The Cancer Genome Atlas (TCGA) dataset. The subtypes were used as labels to build a support vector machine model with cross-validation. We validated the robustness of the model on Chinese Glioma Genome Atlas (CGGA) dataset. DNAm-driven genes were identified by integrating DNAm and gene expression profiling analyses using the R MethylMix package and carried out for further enrichment analysis.

**Results:**

For TCGA dataset, the model produced a high C-index (0.92 ± 0.02), low brier score (0.16 ± 0.02), and significant log-rank *p* value (*p* < 0.0001). The model also had a decent performance for CGGA dataset (CGGA DNAm: C-index of 0.70, brier score of 0.21; CGGA RNA-seq: C-index of 0.79, brier score of 0.18). Moreover, we identified 389 DNAm-driven genes of survival-sensitive subtypes, which were significantly enriched in the glutathione metabolism pathway.

**Conclusions:**

Our study identified two survival-sensitive subtypes of glioma and provided insights into the molecular mechanisms underlying glioma development; thus, potentially providing a new target for the prognostic prediction of gliomas and supporting personalized treatment strategies.

**Supplementary Information:**

The online version contains supplementary material available at 10.1186/s12859-022-04970-x.

## Background

Gliomas are primary brain tumors that arise from differentiated glial cells and include oligodendroglioma, malignant glioma, ependymoma, astrocytoma, oligoastrocytoma, and not otherwise specified [1]. In the United States of America, 45.7% of tumors in children and adolescents are gliomas [2]. Owing to inherent heterogeneity, the prognosis of gliomas varies across different subtypes, with 5-year survival rate of 82.7% for oligodendroglioma and 6.8% for glioblastoma [2]; thus, rendering robust prognosis prediction challenging. Several factors, including age, tumor grade, chemotherapy, and radiotherapy, have been associated with glioma prognosis [3]. Moreover, molecular subtypes have shown distinct differences in survival time*. miR-215* overexpression [4], *miR-637* suppression [5], and *IDH1* wildtype [6] were associated with the poor prognosis of patients with glioma. However, these predictors of prognosis are unstable and greatly influenced by samples selection [7]. We aim to find a new approach for a more accurate glioma prognosis prediction.

Deep learning, a branch of machine learning to model high level abstractions of data using multiple layers of neurons consisting of complex structures [8], has dramatically improved speech recognition, visual object recognition, object detection, and other domains, such as drug discovery and genomics [8]. The advent of deep learning algorithm and the accessibility of multi-omic data represent a new approach for the identification of survival-sensitive subtypes. A recent study which employed a deep learning approach to jointly analyze methylation, miRNA expression, and mRNA expression data, showed improved efficiency in the identification of features linked to survival compared with the use of principal component analysis (PCA) or Cox proportional hazards (Cox-PH) [9]. However, this approach has rarely been used for glioma subtyping.

Herein, using RNA sequencing (RNA-seq) and DNA methylation (DNAm) data from The Cancer Genome Atlas (TCGA), we trained an autoencoder-based model (a deep learning algorithm) to identify survival-sensitive subtypes, and used DNAm-driven genes of subtypes to find the pathways associated with prognosis of gliomas.

## Results

### Identification of two subtypes of gliomas

We preprocessed RNA-seq and DNAm data from TCGA dataset as input features for the autoencoder framework (architecture of autoencoder is shown in Additional file 1). From the bottleneck hidden layer of autoencoder, we obtained 100 new features; 46 of the 100 new features were found to be significantly associated with survival using univariate Cox-PH models (*p* < 0.05). K-means clustering (cluster number ranging from 2 to 6) was applied to the 46 features, and the optimal number of clusters was found to be 2 (calinski harabasz score = 144.58, silhouette score = 0.19; Additional file 2). Thus, we clustered the samples into two subtypes (G1: 346 and G2: 217; Fig. 1); there was significant difference in survival time between the subtypes, with the G2 subtype exhibiting a worse prognosis (log-rank *p* < 0.0001; Fig. 2a).

**Fig. 1:**
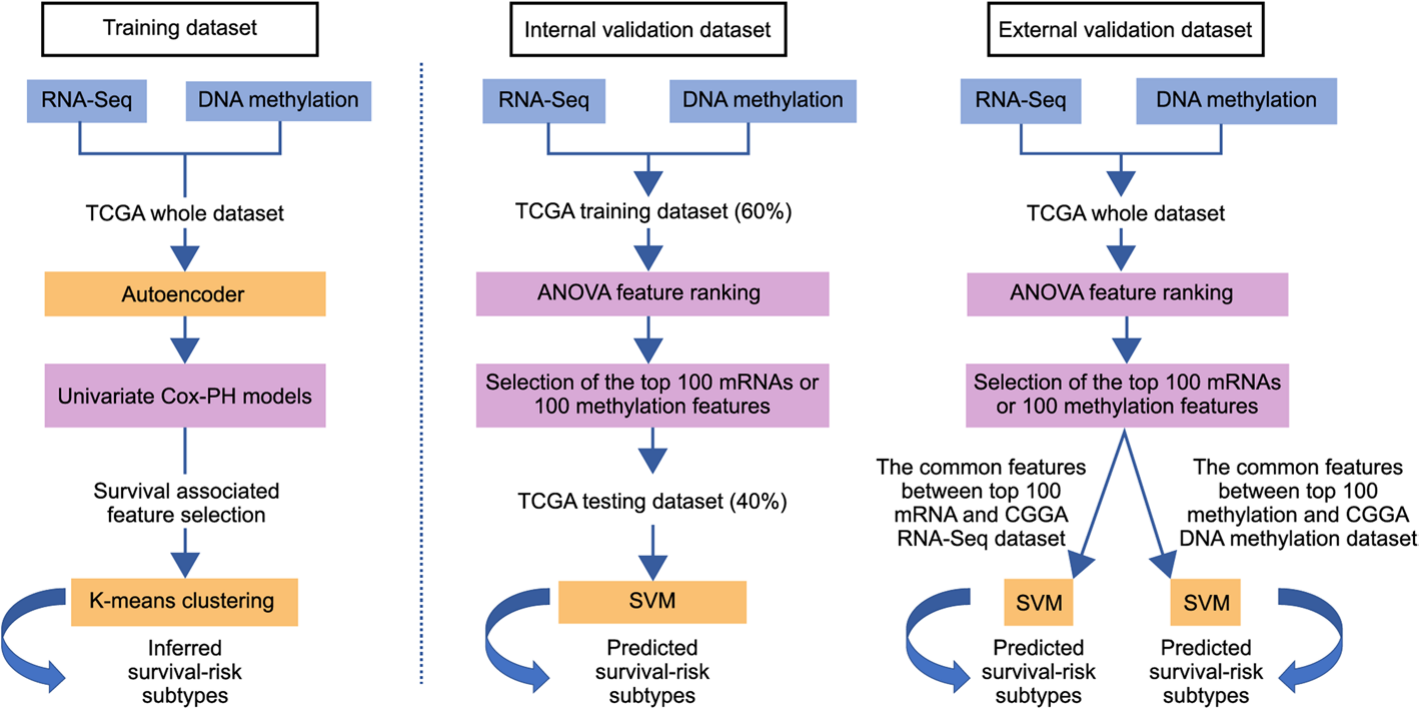
Study design workflow for the identification of glioma subtypes

**Fig. 2:**
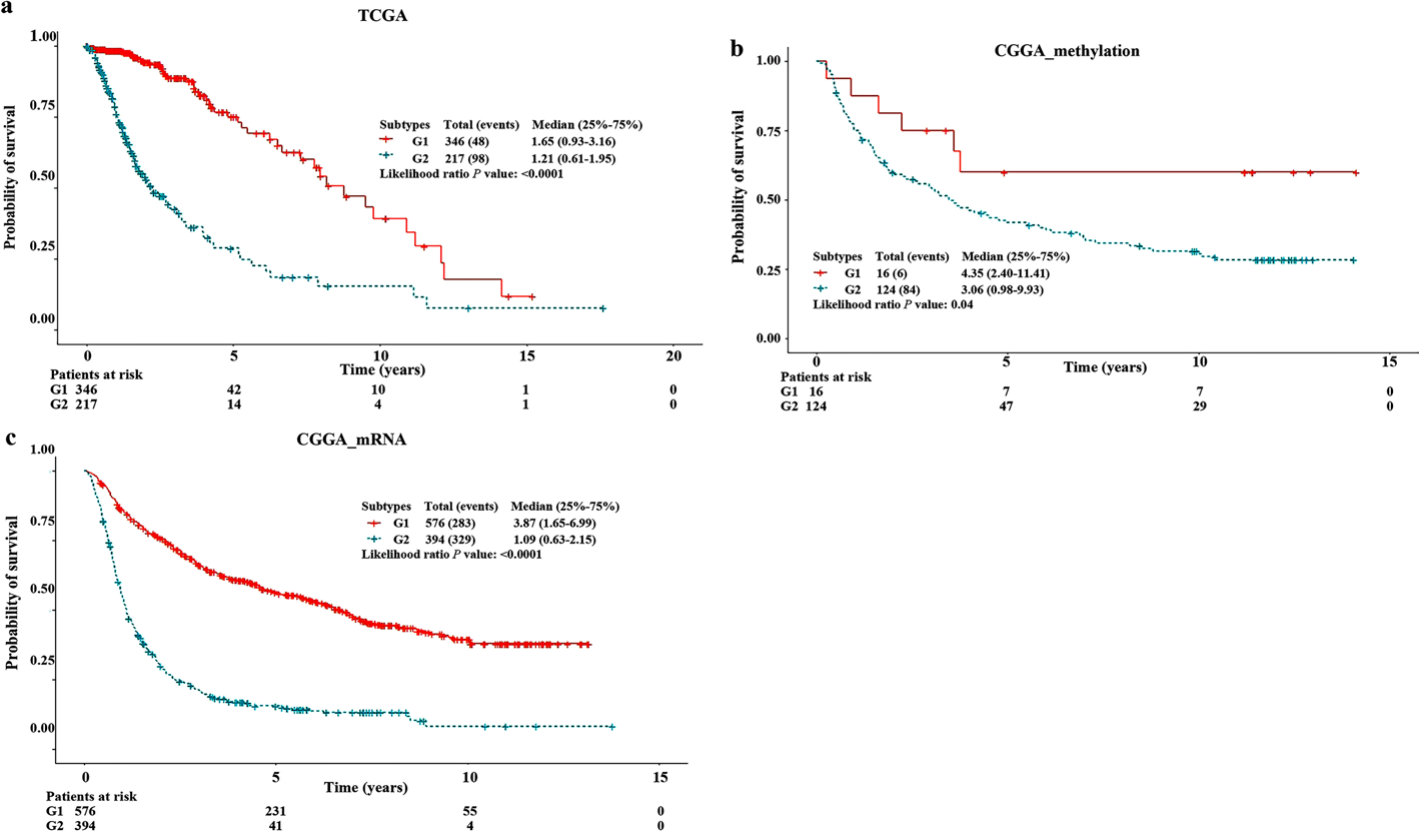
Kaplan–Meier survival curves of the two subtypes in TCGA and CGGA datasets. **a** TCGA dataset. **b** CGGA DNAm dataset. **c** CGGA RNA-seq dataset

### Robustness assessment

To predict on TCGA 2-omics testing dataset, we trained the SVM model from a combination of the top 100 mRNAs and 100 methylation features (Additional file 3). The model produced a high C-index (0.92 ± 0.02), low brier score (0.16 ± 0.02), and significant log-rank *p* value (*p* < 0.0001; Table 1). For the TCGA GBM tumor type, we obtained a C-index of 0.84, brier score of 0.13. As the GBM tumor type had only 56 samples, log-rank *p* value was not significant (*p* = 0.70; Additional file 4). For the TCGA LGG tumor type, we obtained a C-index of 0.90, brier score of 0.16, and significant log-rank *p* value (*p* < 0.0001; Additional file 4). We further predicted on TCGA single omic testing dataset using the corresponding top 100 mRNAs or 100 methylation features. The model also had a decent performance in terms of C-index, brier scores, and log-rank *p* value (Table 1).

**Table 1:** Cross validation-based performance of the SVM model on TCGA testing dataset

Ten folds cross validation	C-index, mean (SD)	Brier score, mean (SD)	Log-rank *p* value, geo.mean
TCGA 2-omics testing dataset (40%)	0.92 (0.02)	0.16 (0.02)	4.68E−12
TCGA mRNA testing dataset	0.92 (0.02)	0.17 (0.02)	3.73E−12
TCGA methylation testing dataset	0.95 (0.02)	0.16 (0.03)	6.33E−13

SVM, Support vector machine; geo.mean, geometric mean

We further used CGGA RNA-seq and CGGA DNAm datasets as external validation datasets (Fig. 1). The number of common features between the top 100 mRNAs and CGGA RNA-seq dataset was 94, and that between the top 100 methylation features and CGGA DNAm dataset was 62. To predict on two external validation datasets, we utilized the common features to build the SVM models. For the CGGA DNAm dataset, we obtained a C-index of 0.70, brier score of 0.21, and significant log-rank *p* value (*p* = 0.04; Table 2, Fig. 2b). For the CGGA RNA-seq dataset, we obtained a C-index of 0.79, brier score of 0.18, and significant log-rank *p* value (*p* < 0.0001; Table 2, Fig. 2c).

**Table 2:** Performance of the SVM model on two external validation datasets

External validation datasets	Samples (N)	C-index	Brier score	Log-rank *p* value
CGGA RNA-seq dataset	970	0.79	0.18	< 0.0001
CGGA DNAm dataset	140	0.70	0.21	0.04

SVM, Support vector machine

### Autoencoder-based approach outperforms alternative approaches

The performance of the autoencoder-based approach was compared with that of the two alternative approaches, PCA and iCluster. Using PCA, we obtained 100 principal components, 29 of which were significantly associated with survival, as determined using univariate Cox-PH models (*p* < 0.05). Moreover, two subtypes were obtained from the 29 principal components via K-means clustering (G1: 562 and G2: 1; Additional file 5). iCluster clustered the samples into two subtypes directly from the initial features (G1: 509 and G2: 54; Additional file 5). Kaplan–Meier survival curves of three approaches are shown in Additional file 6.

The subtypes determined using PCA or iCluster were used as labels to build an SVM model with cross-validation. As the G2 subtype of PCA had only one sample, this approach failed to build the SVM model to predict on the testing dataset. The autoencoder-based approach had a better performance, with a higher C-index (0.92 ± 0.02 vs. 0.90 ± 0.02), than the iCluster approach (Additional file 7).

### Clinical covariate analysis

We examined the statistical differences in clinical covariates between subtypes. The G2 subtype had a higher tumor grade, shorter follow-up time, and higher proportion of GBM type and deaths than the G1 subtype (Additional files 8, 9 and 10). For CGGA RNA-seq datasets, the mutation rate of the *IDH* gene was 51.55%. *IDH* was less frequently mutated in the aggressive G2 subtype than in the G1 subtype (Additional file 9).

To test whether the accuracy of prediction could be improved by adding clinical information, we built a multivariate Cox-PH model (age, gender, tumor types, tumor grade and autoencoder subtypes) and compared the model with autoencoder subtypes (G2 vs. G1) or tumor types (GBM vs. LGG) only model. The model with autoencoder subtypes had better predictive ability than combination model or tumor types model (C-index: 0.91 vs. 0.86 vs. 0.90; Additional file 11).

### Functional analysis of the two glioma subtypes

**Fig. 3:**
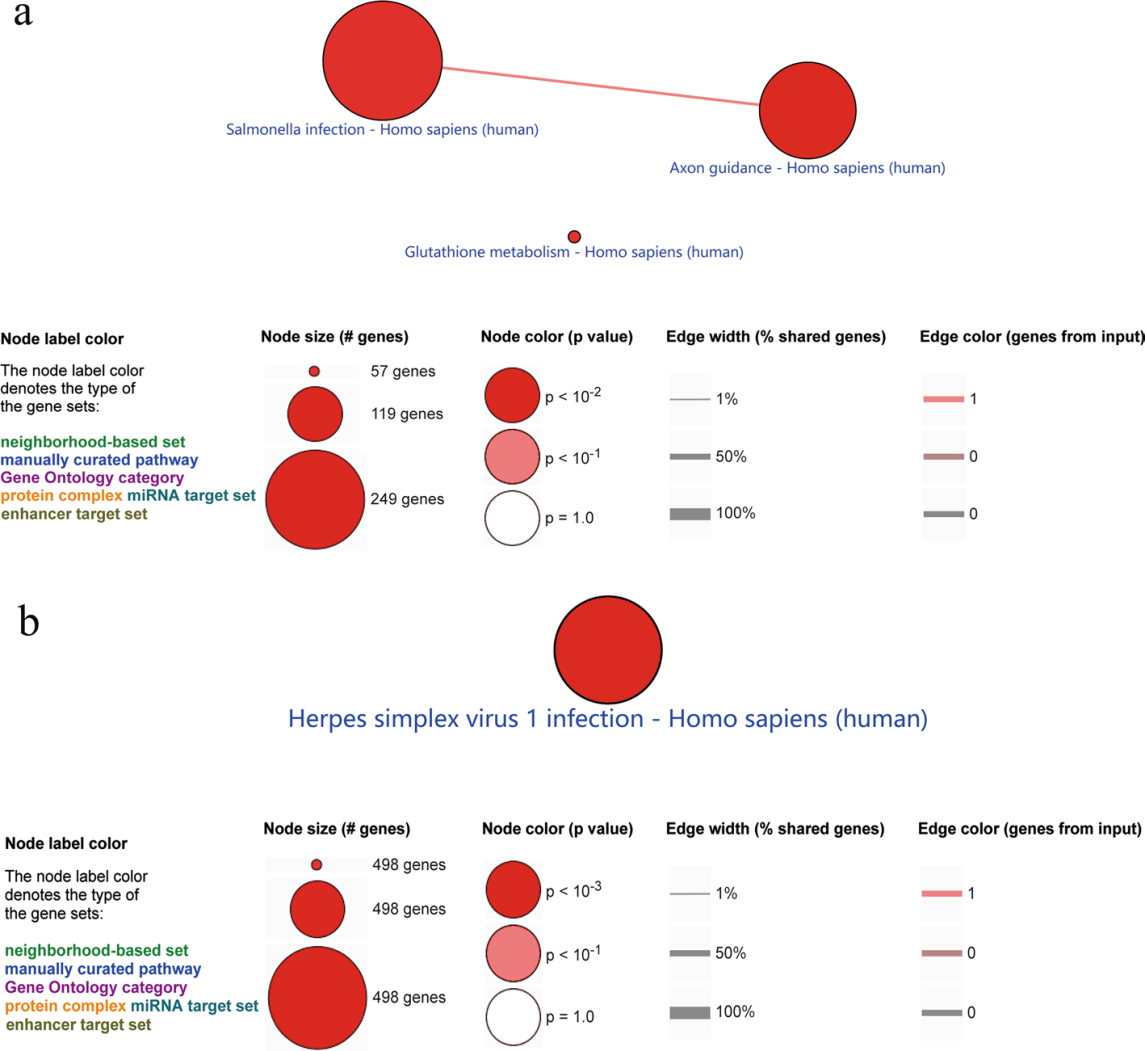
KEGG pathway analysis of DNA methylation driven genes. **a** 305 hypomethylated and highly expressed genes in the G2 subtype. **b** 84 hypermethylated and lowly expressed genes in the G2 subtype

We performed a MethylMix analysis to identify 389 DNAm-driven genes (correlation coefficients r <  − 0.3, Wilcoxon rank-sum tests *p* value < 0.05). Methylation and mRNA levels of DNAm-driven genes were visualized via heatmaps (Additional files 12 and 13). Among these genes, 305 hypomethylated and highly expressed genes in the G2 subtype were significantly enriched in the salmonella infection, axon guidance and glutathione metabolism pathway (*p* < 0.01; Additional file 14, Fig. 3a). Eighty-four hypermethylated and lowly expressed genes in the G2 subtype were significantly enriched in the herpes simplex virus 1 infection pathway (*p* < 0.01; Fig. 3b).

## Discussion

Deep learning is a subgroup of machine learning that has multiple processing layers [10]. This approach has been applied to solve a number of biomedical problems [11], including those associated with image analysis [12], genomics [13], and drug discovery [14]. Matsui et al. [15] trained a deep learning model to jointly analyze magnetic resonance imaging, computed tomography, and positron emission tomography data and identified three subtypes of lower-grade gliomas. The autoencoder, a deep learning algorithm, is capable of jointly learning from multi-omic data without explicitly defining common features [11]. The autoencoder model showed efficiency in the identification of two survival-sensitive subtypes of neuroblastoma [16]. However, this approach has rarely been used for glioma subtyping.

Herein, we demonstrated that the autoencoder-based approach could capture core prognostic features and effectively identify two survival-sensitive subtypes of glioma. First, cross-validation results revealed a decent performance in TCGA testing dataset. Second, this model was validated in two external validation datasets, CGGA RNA-seq and CGGA DNAm. Third, the model showed increased efficiency in the identification of features relevant to the prognosis than PCA or iCluster. Finally, the performance of the model was not improved upon the addition of clinical information.

We also explored molecular subtypes and biological pathways involved in the prognosis of glioma. In concordance with a previous study [6], *IDH* was less frequently mutated in the aggressive G2 subtype than in the G1 subtype. Moreover, we identified 389 DNAm-driven genes, and found that 305 hypomethylated and highly expressed genes in the G2 subtype were significantly enriched in the glutathione metabolism pathway. It has been reported that the imbalance of glutamate homeostasis in the central nervous system is related to the occurrence and development of gliomas. Glioma cells can release a large amount of glutamate, transport glutamate to the outside of the cell through the glutamate/cystine transporter, and take cystine into the cell to synthesize glutathione to increase the antioxidant capacity of tumor cells [17]. Pharmacologic inhibition of the nuclear factor erythroid 2-related factor 2/glutathione pathway via brusatol administration exhibited a potent tumor suppressive effect on *IDH1*-mutated glioma in vitro and in vivo [18].

There are several limitations in this study. First, more validation datasets are necessary for demonstrating the robustness of the model. Second, the clinical covariates of patients are not always known in public datasets, restricting our confirmation effort.

## Conclusions

Our study identifies two survival-sensitive subtypes of glioma and provides insights into the molecular mechanisms underlying glioma development; thus, potentially providing a new target for the prognostic prediction of gliomas and supporting personalized treatment strategies.

## Methods

### Data extraction and normalization

Data were obtained from TCGA and Chinese Glioma Genome Atlas (CGGA) datasets. TCGA, a project to understand the molecular mechanisms of cancer, has data on 1122 glioma samples [19]; 563 samples with 25,292 genes from RNA-seq and 18,976 genes from DNAm data were used as the training dataset. CGGA, a project to investigate brain tumors, has data on 2000 glioma samples collected from Chinese cohorts [20]; 970 samples with 23,271 genes from RNA-seq and 140 samples with 14,476 genes from DNAm data were used as external validation datasets.

We applied 2-step normalization on both training and validation datasets [21]. First, we used the median absolute deviation on both the training and validation datasets. Second, we applied the robust scale normalization on the training dataset, and scaled the validation dataset using the means and standard deviations of the training dataset (Additional file 15).

### Construction of an autoencoder model

The autoencoder algorithm is a reduction method implemented using artificial neural networks. We used autoencoder to reconstruct $x$ by the output ${x}^{{\prime}}$. Tanh was used as the activation function for the $i$ layer [9], that is:

$$\gamma ={f}_{i}\left(x\right)=\mathrm{tanh}({W}_{i}.x+{b}_{i})$$

where $x$ and $\gamma$ are two vectors of size d and p; ${W}_{i}$ is the weight matrix of size p × d; ${b}_{i}$ is an intercept vector of size p; and ${W}_{i}.x$ gives a vector of size p [9].

For a k-layer autoencoder model, ${x}^{{\prime}}$ is:

$${x}^{{\prime}}={F}_{1\to k}\left(x\right)={f}_{1}\dots {f}_{k-1}{f}_{k}(x)$$

Logloss was used as the loss function to assess the error between $x$ and ${x}^{{\prime}}$ [9], that is:

$$logloss\left(x,{x}^{{\prime}}\right)=\sum_{k=1}^{d}({x}_{k}\mathrm{log}\left({x}_{k}^{{\prime}}\right)+\left(1-{x}_{k}\right)\mathrm{log}\left(1-{x}_{k}^{{\prime}}\right))$$

To control overfitting, L1 regularization penalty ${a}_{w}$ was added on ${W}_{i}$, and L2 regularization penalty ${a}_{a}$ was added on ${F}_{1\to i}\left(x\right)$ [9], that is:

$$L\left(x,{x}^{{\prime}}\right)={\textit{logloss}}\left(x,{x}^{{\prime}}\right)+\sum_{i=1}^{k}({{a}_{w}||{w}_{i}||}_{1}+{{a}_{a}||{F}_{1\to i}\left(x\right)||}_{2}^{2})$$

We used preprocessed data from TCGA dataset as the input for the autoencoder framework. We constructed a five-layer autoencoder model with three hidden layers (500, 100, and 500 nodes). The bottleneck layer was used to obtain 100 new features. We set the L1 regularization to 0.0001 and L2 regularization to 0.001. The autoencoder was trained using a gradient descent algorithm with 10 epochs and 50% dropout, a learning rate of 1E-06, and a batch size of 32 (using the PythonKeras library).

### Feature selection and K-means clustering

Survival-associated features were selected from the 100 new features using univariate Cox-PH models (*p* < 0.05, using the R survival package). The labels for different subtypes were obtained via K-means clustering from survival-associated features (using the Python scikit-learn package). We determined the optimal number of clusters using the calinski harabasz score and silhouette score.

### Robustness assessment

We demonstrated the robustness of the model using internal and external validation datasets. After obtaining the labels, we built a support vector machine (SVM) model with cross-validation. The 563 samples of TCGA dataset were split into 10 folds for model training and testing with a 6/4 ratio. We selected the top 100 mRNAs or 100 methylation features of TCGA training dataset based on analysis of variance (ANOVA) F values. To predict on TCGA 2-omics testing dataset, we trained the SVM model from a combination of the top 100 mRNAs and 100 methylation features selected above. We further predicted on TCGA single omic testing dataset using the corresponding top 100 mRNAs or 100 methylation features.

We further used CGGA RNA-seq and CGGA DNAm datasets as external validation datasets. To predict on two external validation datasets, we utilized the common features between the top 100 mRNAs or 100 methylation features of the whole TCGA dataset and CGGA dataset to build the SVM model.

### Evaluation metrics

We used three metrics to reflect the accuracy of survival prediction of the model. Log-rank *p* value, used to evaluate the survival difference between subgroups [22], and concordance index (C-index), used to assess the predictive ability of the model [23], were calculated using the R survival package. Brier score, used to measure the accuracy of probabilistic prediction, was calculated using the Python scikit-learn package [24].

### Two alternative approaches

We further compared the performance of autoencoder-based approach with PCA and iCluster using the data from TCGA dataset. One hundred principal components were obtained by PCA (using the Python scikit-learn package), which was the same number as features in the bottleneck layer of autoencoder. Survival-associated principal components were selected from the 100 principal components using univariate Cox-PH models (*p* < 0.05, using the R survival package). Labels were obtained via K-means clustering from survival-associated principal components (using the Python scikit-learn package). iCluster obtained labels directly from initial features (using the R iCluster package) [25]. After obtaining the labels, we also built SVM models with cross-validation. The performance of the model was evaluated using the above three metrics.

### Clinical covariate analysis

We examined the statistical differences in clinical covariates (age, gender, tumor grade, tumor type) between autoencoder subtypes using Wilcoxon rank-sum tests for continuous variables and χ^2^ tests for categorical variables. To test whether the accuracy of prediction could be improved by adding clinical information, we built a multivariate Cox-PH model (age, gender, tumor grade, tumor type and autoencoder subtypes) and compared the model with autoencoder subtypes or tumor types only model.

A systematic review reported that the *IDH1* mutation is an independent factor for longer overall survival in patients with glioblastoma [6]. We performed χ^2^ tests on the *IDH* mutation between subtypes from the CGGA RNA-seq dataset.

### Functional analysis

Functional analyses were performed to understand the characteristics of the autoencoder subtypes from TCGA dataset. DNAm-driven genes were identified by integrating DNAm and gene expression profiling analyses using the R MethylMix package [26]. DNAm-driven genes met the following two conditions: (1) DNAm levels of these genes were negatively correlated with the mRNA expression levels. The correlation coefficient was calculated using Spearman’s correlation test (correlation coefficients r <  − 0.3). (2) There were significant differences in the levels of DNAm between autoencoder subtypes (Wilcoxon rank-sum tests *p* value < 0.05). Kyoto Encyclopedia of Genes and Genomes (KEGG) pathway analysis was performed to determine the enriched pathways of DNAm-driven genes (*p* < 0.01). The results of KEGG pathway analysis were visualized via ConsensusPathDB (http://cpdb.molgen.mpg.de/).

## Supplementary Information

**Additional file 1: Figure S1**. Architecture of the autoencoder.

**Additional file 2: Figure S2**. Selection of the optimal number of clusters.

**Additional file 3: Table S1**. Top 100 mRNAs or 100 methylation features of the whole TCGA dataset.

**Additional file 4: Table S2**. Performance of the SVM model on tumor types.

**Additional file 5: Table S3**. Subtypes identified using alternative approaches.

**Additional file 6: Figure S3**. Kaplan-Meier survival curves of three approaches. (a) Autoencoder-based approach. (b) iCluster. (c) PCA.

**Additional file 7: Table S4**. Cross validation-based performance of the SVM model when using alternative approaches.

**Additional file 8: Table S5**. Clinical information of TCGA dataset.

**Additional file 9: Table S6**. Clinical information of CGGA RNA-seq dataset.

**Additional file 10: Table S7**. Clinical information of CGGA DNAm dataset.

**Additional file 11: Table S8**. Performance of the model based on clinical features.

**Additional file 12: Figure S4**. mRNA levels of DNA methylation driven genes.

**Additional file 13: Figure S5**. Methylation levels of DNA methylation driven genes.

**Additional file 14: Table S9**. KEGG pathway analysis of DNAm-driven genes.

**Additional file 15**: Supplementary methods. Description of normalization methods used data normalization.

## Data Availability

Data analyzed in this study were obtained from TCGA (https://portal.gdc.cancer.gov/repository. DataSet ID: TCGA-GBM Transcriptome Profiling and TCGA-GBM DNA Methylation TCGA-LGG Transcriptome Profiling and TCGA-LGG DNA Methylation) and CGGA (http://www.cgga.org.cn/download.jsp. DataSet ID: mRNAseq_693, mRNAseq_325 and methyl_159). Softwares, a test dataset and the code described in the paper were accessible from github (https://github.com/Deep-learning-omics/autoencoder).

## Abbreviations

CGGA: Chinese Glioma Genome Atlas
C-index: Concordance index
Cox-PH: Cox proportional hazards
DEGs: Differentially expressed genes
DNAm: DNA methylation
FDR: False discovery rate
GBM: Glioblastoma
KEGG: Kyoto Encyclopedia of Genes and Genomes
LGG: Low grade glioma
PCA: Principal component analysis
RNA-seq: RNA sequencing
SVM: Support vector machine
TCGA: The Cancer Genome Atlas
