# Supplementary material for: Deep learning algorithm reveals two prognostic subtypes in patients with gliomas
Source: BMC Bioinformatics. 2022 Oct 11;23:417. doi: 10.1186/s12859-022-04970-x (PMC9552440; doi:10.1186/s12859-022-04970-x)
Supplement: Supplementary file 1 — Additional file 1: Figure S1. Architecture of the autoencoder. [file 12859_2022_4970_MOESM1_ESM.docx]

**Supplementary Files**

**Additional File 1**

**Figure S1**. Architecture of the autoencoder


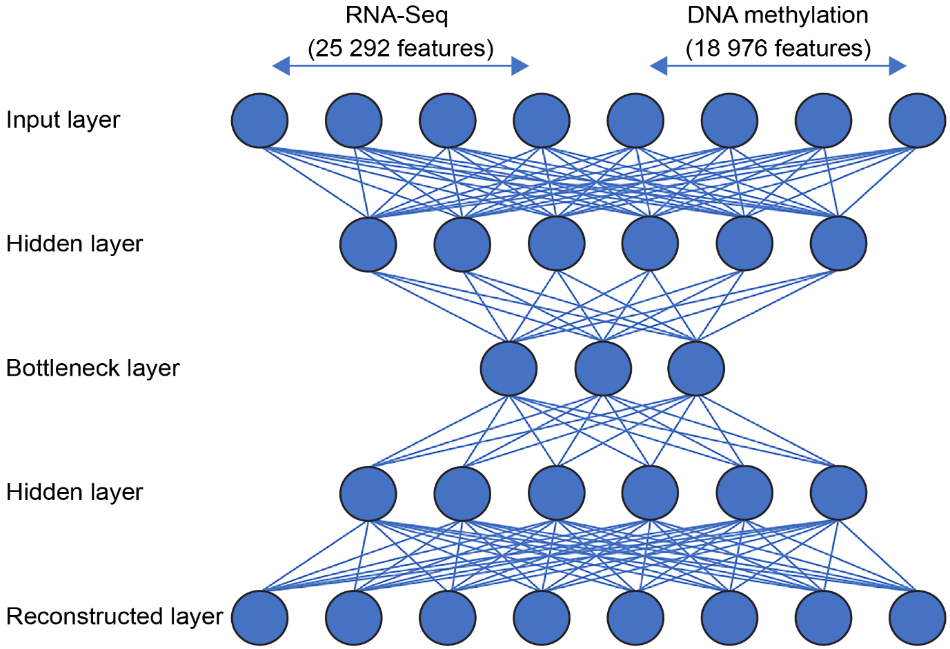
Figure S1. Architecture of the autoencoder
