# Supplementary figures and images for: Deep learning algorithm reveals two prognostic subtypes in patients with gliomas
Source: BMC Bioinformatics. 2022 Oct 11;23:417. doi: 10.1186/s12859-022-04970-x (PMC9552440; doi:10.1186/s12859-022-04970-x)

**Supplementary Files**

**Additional File 2**

**Figure S2**. Selection of the optimal number of clusters


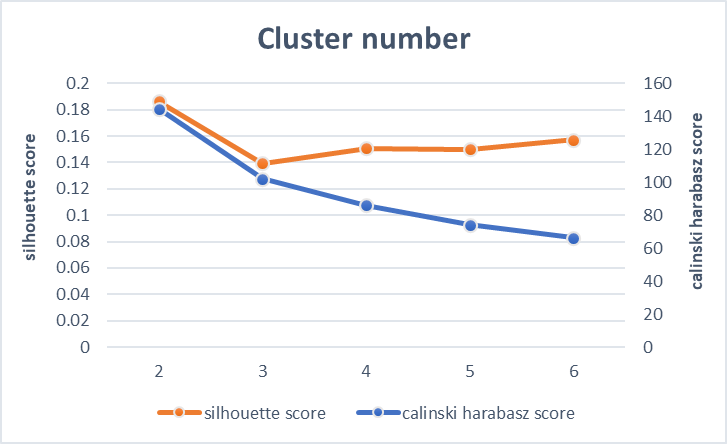

Supplement: Supplementary file 2 — Additional file 2: Figure S2. Selection of the optimal number of clusters. [file 12859_2022_4970_MOESM2_ESM.docx]
