# Supplementary material for: Deep learning algorithm reveals two prognostic subtypes in patients with gliomas
Source: BMC Bioinformatics. 2022 Oct 11;23:417. doi: 10.1186/s12859-022-04970-x (PMC9552440; doi:10.1186/s12859-022-04970-x)
Supplement: Supplementary file 3 — Additional file 3: Table S1. Top 100 mRNAs or 100 methylation features of the whole TCGA dataset. [file 12859_2022_4970_MOESM3_ESM.docx]

**Supplementary Files**

**Additional File 3**

**Table S1**. Top 100 mRNAs or 100 methylation features of the whole TCGA dataset

| mRNA features | *p* value | Methylation features | *p* value |
| --- | --- | --- | --- |
| *FBXO25* | 3.16E-55 | *PTDSS2* | 1.81E-78 |
| *KDELR1* | 2.23E-53 | *PCGF3* | 4.92E-77 |
| *TRIP6* | 6.45E-61 | *MAP7D1* | 4.22E-77 |
| *SYDE1* | 6.51E-51 | *OAS2* | 4.51E-74 |
| *HDAC4* | 7.15E-53 | *TAP2* | 6.66E-76 |
| *ATAD1* | 5.18E-54 | *ERMAP* | 8.36E-76 |
| *ZNF25* | 5.27E-56 | *WISP3* | 5.81E-73 |
| *CLASP2* | 7.39E-62 | *WHSC1* | 1.04E-78 |
| *SLC25A21-AS1* | 5.96E-52 | *BRD2* | 4.12E-74 |
| *ALDH5A1* | 5.29E-58 | *MIR125B2* | 3.23E-73 |
| *SWAP70* | 7.88E-54 | *WDR88* | 1.08E-76 |
| *RNF135* | 1.21E-63 | *CLCN7* | 3.17E-73 |
| *XKR8* | 8.25E-51 | *SRRM2* | 3.73E-74 |
| *AL157392.3* | 6.76E-53 | *EZR* | 5.10E-78 |
| *AC009227.1* | 2.79E-55 | *ATP6V1G2-DDX39B* | 9.56E-81 |
| *TBC1D1* | 5.42E-54 | *ASH1L* | 2.88E-76 |
| *EFEMP2* | 1.14E-55 | *AACS* | 9.37E-74 |
| *AC120036.4* | 1.42E-65 | *RP4-569M23.5* | 2.93E-81 |
| *NMI* | 2.11E-50 | *PC* | 1.35E-74 |
| *SCAPER* | 3.52E-52 | *TBCD* | 2.38E-76 |
| *DNM3* | 2.72E-56 | *IRF9* | 2.32E-76 |
| *FUCA2* | 1.50E-53 | *DBN1* | 1.92E-75 |
| *SHISA7* | 6.31E-54 | *ZSWIM8* | 1.71E-73 |
| *FBXO17* | 1.55E-57 | *MICAL3* | 2.70E-74 |
| *ZDHHC12* | 1.41E-56 | *RP11-66N24.4* | 7.99E-75 |
| *PHYHIPL* | 4.87E-51 | *SLC45A1* | 1.51E-73 |
| *ZNF248* | 4.39E-54 | *TPST2* | 5.40E-78 |
| *MSN* | 3.29E-72 | *TRAPPC12* | 6.86E-74 |
| *TMBIM1* | 1.73E-50 | *LINC00925* | 1.56E-75 |
| *PRICKLE3* | 5.91E-57 | *KIAA0195* | 1.30E-77 |
| *SP140L* | 7.05E-57 | *CSNK2B* | 1.60E-73 |
| *ANKRD16* | 1.53E-51 | *MDK* | 1.87E-78 |
| *FAM114A1* | 2.99E-53 | *CIITA* | 4.65E-76 |
| *FBXL20* | 3.24E-51 | *TMEM247* | 1.22E-74 |
| *SLC2A10* | 1.50E-57 | *FAM57B* | 3.31E-80 |
| *POU6F1* | 6.82E-52 | *KAT6B* | 2.56E-75 |
| *GLA* | 1.02E-52 | *SMARCA4* | 2.56E-80 |
| *CEP112* | 4.21E-62 | *DOK7* | 7.77E-74 |
| *APOBEC3F* | 1.34E-56 | *KIAA0930* | 1.50E-80 |
| *ADPRH* | 1.51E-57 | *PARVB* | 2.22E-78 |
| *CGAS* | 8.76E-51 | *VARS* | 1.33E-73 |
| *GSDMD* | 3.75E-54 | *DLGAP4-AS1* | 1.46E-76 |
| *EVC2* | 3.30E-52 | *VPS51* | 5.89E-75 |
| *ZNF33A* | 6.87E-54 | *AC005808.3* | 9.04E-76 |
| *FRY* | 3.89E-55 | *AL513523.2* | 2.26E-77 |
| *GNG12* | 2.67E-55 | *HRH1* | 1.22E-76 |
| *FCHSD1* | 6.01E-53 | *ZCCHC14* | 2.83E-73 |
| *CASP6* | 2.87E-69 | *ITGAX* | 7.64E-86 |
| *ARMCX4* | 1.91E-51 | *OGG1* | 8.82E-76 |
| *ANXA5* | 8.43E-53 | *ABCA2* | 2.00E-74 |
| *FCHSD2* | 1.18E-57 | *ABCA4* | 2.12E-73 |
| *FHL3* | 4.23E-52 | *TCF3* | 6.63E-75 |
| *RBSN* | 8.35E-65 | *LRP12* | 1.14E-75 |
| *DCTD* | 3.78E-68 | *KCTD9P1* | 8.14E-77 |
| *TUB* | 1.42E-62 | *CHFR* | 1.49E-77 |
| *GNG5* | 6.97E-53 | *SART1* | 4.09E-74 |
| *ATP5S* | 1.54E-52 | *STK25* | 2.46E-75 |
| *FAM155A* | 1.37E-51 | *H3F3AP6* | 1.12E-75 |
| *CTIF* | 1.38E-52 | *GGA2* | 1.05E-75 |
| *ANKRD26* | 7.90E-54 | *AC087651.1* | 1.92E-77 |
| *REEP4* | 1.82E-51 | *KMT2C* | 2.31E-73 |
| *REXO2* | 1.09E-50 | *BNIPL* | 1.89E-73 |
| *PDZD8* | 8.04E-53 | *RP5-1047A19.4* | 8.19E-75 |
| *BCL2L12* | 2.43E-57 | *FAM160B2* | 4.91E-73 |
| *CNRIP1* | 9.58E-52 | *RHBDD2* | 3.92E-73 |
| *GALM* | 3.22E-53 | *CNOT1* | 8.36E-75 |
| *SDF4* | 2.08E-51 | *LIF* | 1.48E-79 |
| *RAB34* | 4.72E-52 | *USP49* | 2.62E-75 |
| *RIPK1* | 1.20E-53 | *SLC4A10* | 1.65E-75 |
| *TTC3* | 9.24E-53 | *C6orf136* | 3.10E-73 |
| *IQGAP1* | 2.03E-52 | *LPIN1* | 2.14E-74 |
| *ADAM22* | 4.16E-53 | *TMEM106A* | 5.95E-75 |
| *CCSER2* | 4.16E-62 | *LMF2* | 3.60E-73 |
| *GSAP* | 1.97E-61 | *CYTH1* | 6.61E-76 |
| *SPATS2L* | 1.63E-57 | *JAG1* | 7.22E-76 |
| *GDPD1* | 2.23E-51 | *NAA40* | 6.53E-81 |
| *ARSD* | 2.61E-54 | *SPG7* | 2.66E-74 |
| *ARPP21* | 5.69E-55 | *TUBB2A* | 1.68E-73 |
| *CSMD3* | 4.55E-52 | *KLHL2* | 5.76E-75 |
| *SLC43A3* | 2.14E-55 | *ANKRD6* | 9.19E-78 |
| *NDUFA6-DT* | 1.26E-52 | *AC009505.2* | 2.18E-74 |
| *ZMYND11* | 4.08E-51 | *AP003419.11* | 9.98E-96 |
| *MAPT* | 5.22E-70 | *PPM1M* | 3.03E-81 |
| *BCL7A* | 9.00E-63 | *FMOD* | 3.61E-75 |
| *SP100* | 2.87E-52 | *RAC2* | 3.56E-80 |
| *WAC* | 1.14E-56 | *FOXK2* | 2.83E-73 |
| *TNRC6C* | 1.92E-52 | *CPSF1* | 3.24E-73 |
| *B3GNT5* | 4.20E-51 | *PRDM15* | 3.10E-76 |
| *MAPK8* | 1.20E-57 | *NF1* | 2.52E-79 |
| *SLC39A1* | 1.11E-53 | *TAGLN2* | 5.36E-73 |
| *PKMP3* | 9.28E-52 | *NINJ1* | 7.96E-75 |
| *TGIF1* | 7.93E-60 | *TYK2* | 4.67E-75 |
| *CASP8* | 9.79E-66 | *MIR33A* | 9.13E-74 |
| *MICU1* | 2.72E-52 | *CTD-2207O23.12* | 1.19E-75 |
| *LINC00672* | 8.03E-52 | *NFATC4* | 6.17E-83 |
| *SLC30A7* | 2.26E-51 | *SLC1A5* | 3.61E-76 |
| *C9orf64* | 5.90E-59 | *ATP6V1G2* | 9.17E-74 |
| *APOBEC3G* | 3.39E-53 | *PVRL1* | 9.02E-76 |
| *TP73-AS1* | 4.49E-63 | *ZSCAN5A* | 4.60E-76 |
| *SLITRK5* | 1.02E-51 | *PIGG* | 8.17E-79 |
