# Supplementary material for: Deep learning algorithm reveals two prognostic subtypes in patients with gliomas
Source: BMC Bioinformatics. 2022 Oct 11;23:417. doi: 10.1186/s12859-022-04970-x (PMC9552440; doi:10.1186/s12859-022-04970-x)
Supplement: Supplementary file 4 — Additional file 4: Table S2. Performance of the SVM model on tumor types. [file 12859_2022_4970_MOESM4_ESM.docx]

**Supplementary Files**

**Additional File 4**

**Table S2**. Performance of the SVM model on tumor types

| Tumor types | C-index | Brier score | Log-rank *p* value |
| --- | --- | --- | --- |
| GBM | 0.84 | 0.13 | 0.70 |
| LGG | 0.90 | 0.16 | 4e-16 |

SVM, support vector machine.
