# Supplementary material for: Deep learning algorithm reveals two prognostic subtypes in patients with gliomas
Source: BMC Bioinformatics. 2022 Oct 11;23:417. doi: 10.1186/s12859-022-04970-x (PMC9552440; doi:10.1186/s12859-022-04970-x)
Supplement: Supplementary file 5 — Additional file 5: Table S3. Subtypes identified using alternative approaches. [file 12859_2022_4970_MOESM5_ESM.docx]

**Supplementary Files**

**Additional File 5**

**Table S3**. Subtypes identified using alternative approaches

| ID | PCA | iCluster | Autoencoder |
| --- | --- | --- | --- |
| TCGA-HT-7902 | G1 | G1 | G1 |
| TCGA-VM-A8CA | G1 | G1 | G1 |
| TCGA-DU-6396 | G1 | G1 | G2 |
| TCGA-E1-A7YM | G1 | G1 | G2 |
| TCGA-E1-5318 | G1 | G1 | G1 |
| TCGA-FG-5962 | G1 | G1 | G1 |
| TCGA-HT-7695 | G1 | G1 | G1 |
| TCGA-S9-A6TY | G1 | G1 | G1 |
| TCGA-DU-6401 | G1 | G1 | G1 |
| TCGA-P5-A77W | G1 | G1 | G1 |
| TCGA-P5-A72Z | G1 | G1 | G1 |
| TCGA-FG-A87Q | G1 | G1 | G2 |
| TCGA-HW-7489 | G1 | G1 | G1 |
| TCGA-HT-8105 | G1 | G1 | G1 |
| TCGA-CS-6290 | G1 | G1 | G1 |
| TCGA-TM-A84J | G1 | G1 | G2 |
| TCGA-FG-A6J3 | G1 | G1 | G1 |
| TCGA-E1-A7YL | G1 | G1 | G2 |
| TCGA-HT-7481 | G1 | G1 | G1 |
| TCGA-DB-5270 | G1 | G1 | G1 |
| TCGA-S9-A7IX | G1 | G1 | G2 |
| TCGA-TQ-A7RF | G1 | G1 | G2 |
| TCGA-CS-6666 | G1 | G1 | G1 |
| TCGA-HT-8010 | G1 | G1 | G1 |
| TCGA-DU-8163 | G1 | G1 | G1 |
| TCGA-DU-6397 | G1 | G1 | G1 |
| TCGA-HT-7611 | G1 | G1 | G1 |
| TCGA-TQ-A7RW | G1 | G1 | G2 |
| TCGA-S9-A6WH | G1 | G1 | G1 |
| TCGA-DB-A64U | G1 | G1 | G1 |
| TCGA-TM-A84B | G1 | G1 | G2 |
| TCGA-S9-A6UB | G1 | G1 | G1 |
| TCGA-QH-A65Z | G1 | G1 | G1 |
| TCGA-S9-A6WD | G1 | G1 | G1 |
| TCGA-HT-A5R7 | G1 | G1 | G2 |
| TCGA-WH-A86K | G1 | G1 | G1 |
| TCGA-S9-A6WE | G1 | G1 | G1 |
| TCGA-HT-7692 | G1 | G1 | G1 |
| TCGA-E1-5322 | G1 | G1 | G1 |
| TCGA-DU-6395 | G1 | G1 | G1 |
| TCGA-RY-A847 | G1 | G1 | G1 |
| TCGA-14-1034 | G1 | G2 | G2 |
| TCGA-S9-A7R3 | G1 | G1 | G1 |
| TCGA-DU-A6S3 | G1 | G1 | G2 |
| TCGA-HT-A5R9 | G1 | G1 | G1 |
| TCGA-06-5410 | G1 | G2 | G2 |
| TCGA-DU-A5TW | G1 | G1 | G1 |
| TCGA-06-5856 | G1 | G2 | G2 |
| TCGA-HT-A618 | G1 | G1 | G2 |
| TCGA-HT-8011 | G1 | G1 | G2 |
| TCGA-DH-A7UU | G1 | G1 | G1 |
| TCGA-S9-A6WN | G1 | G1 | G2 |
| TCGA-VV-A829 | G1 | G1 | G1 |
| TCGA-DU-A7TI | G1 | G1 | G2 |
| TCGA-FG-A6IZ | G1 | G1 | G1 |
| TCGA-HT-7881 | G1 | G1 | G1 |
| TCGA-DU-6404 | G1 | G1 | G2 |
| TCGA-HW-7493 | G1 | G1 | G2 |
| TCGA-FG-8182 | G1 | G1 | G1 |
| TCGA-P5-A736 | G1 | G1 | G2 |
| TCGA-CS-6669 | G1 | G1 | G1 |
| TCGA-HT-8111 | G1 | G1 | G1 |
| TCGA-S9-A6TZ | G1 | G1 | G1 |
| TCGA-DB-5275 | G1 | G1 | G1 |
| TCGA-P5-A5EX | G1 | G1 | G2 |
| TCGA-DU-A5TS | G1 | G1 | G2 |
| TCGA-TQ-A7RH | G1 | G1 | G2 |
| TCGA-DB-5277 | G1 | G1 | G1 |
| TCGA-76-4925 | G1 | G2 | G2 |
| TCGA-S9-A6TW | G1 | G1 | G1 |
| TCGA-KT-A74X | G1 | G1 | G1 |
| TCGA-VW-A8FI | G1 | G1 | G2 |
| TCGA-06-5858 | G1 | G2 | G2 |
| TCGA-HT-7605 | G1 | G1 | G1 |
| TCGA-19-1389 | G1 | G2 | G2 |
| TCGA-HT-A74K | G1 | G1 | G2 |
| TCGA-HT-7604 | G1 | G1 | G1 |
| TCGA-HT-7684 | G1 | G1 | G2 |
| TCGA-TM-A84L | G1 | G1 | G2 |
| TCGA-S9-A6U2 | G1 | G1 | G1 |
| TCGA-DU-A6S2 | G1 | G1 | G1 |
| TCGA-DB-A4X9 | G1 | G1 | G1 |
| TCGA-CS-5393 | G1 | G1 | G1 |
| TCGA-DU-6402 | G1 | G1 | G2 |
| TCGA-TQ-A7RM | G1 | G1 | G1 |
| TCGA-HT-7693 | G1 | G1 | G1 |
| TCGA-DU-A76O | G1 | G1 | G1 |
| TCGA-S9-A6WI | G1 | G1 | G1 |
| TCGA-CS-6188 | G1 | G1 | G2 |
| TCGA-HT-7858 | G1 | G1 | G1 |
| TCGA-CS-6670 | G1 | G1 | G1 |
| TCGA-28-5215 | G1 | G2 | G2 |
| TCGA-26-5139 | G1 | G2 | G2 |
| TCGA-DU-6394 | G1 | G1 | G1 |
| TCGA-CS-5396 | G1 | G1 | G1 |
| TCGA-DB-A64X | G1 | G1 | G1 |
| TCGA-DB-A4XH | G1 | G1 | G1 |
| TCGA-QH-A6CW | G1 | G1 | G1 |
| TCGA-S9-A7R1 | G1 | G1 | G1 |
| TCGA-E1-5305 | G1 | G1 | G1 |
| TCGA-DU-8164 | G1 | G1 | G1 |
| TCGA-28-5218 | G1 | G2 | G2 |
| TCGA-HT-7616 | G1 | G1 | G2 |
| TCGA-28-5213 | G1 | G2 | G2 |
| TCGA-06-5417 | G1 | G2 | G2 |
| TCGA-E1-A7YW | G1 | G1 | G2 |
| TCGA-32-1980 | G1 | G2 | G2 |
| TCGA-HT-7476 | G1 | G1 | G1 |
| TCGA-DU-7013 | G1 | G1 | G2 |
| TCGA-FG-7634 | G1 | G1 | G1 |
| TCGA-DU-7015 | G1 | G1 | G2 |
| TCGA-FG-8181 | G1 | G1 | G1 |
| TCGA-DU-8165 | G1 | G1 | G2 |
| TCGA-P5-A5F6 | G1 | G1 | G2 |
| TCGA-FG-6688 | G1 | G1 | G2 |
| TCGA-S9-A7QZ | G1 | G1 | G1 |
| TCGA-DU-7306 | G1 | G1 | G1 |
| TCGA-06-0211 | G1 | G2 | G2 |
| TCGA-TM-A84Q | G1 | G1 | G1 |
| TCGA-TM-A7CA | G1 | G1 | G1 |
| TCGA-HT-A4DV | G1 | G1 | G1 |
| TCGA-FG-A70Z | G1 | G1 | G2 |
| TCGA-HT-7855 | G1 | G1 | G1 |
| TCGA-HT-7879 | G1 | G1 | G2 |
| TCGA-FG-A4MU | G1 | G1 | G2 |
| TCGA-HT-8018 | G1 | G1 | G2 |
| TCGA-HT-7880 | G1 | G1 | G1 |
| TCGA-DU-A6S7 | G1 | G1 | G1 |
| TCGA-HT-A5RB | G1 | G1 | G1 |
| TCGA-QH-A6CV | G1 | G1 | G2 |
| TCGA-DU-7309 | G1 | G1 | G1 |
| TCGA-76-4931 | G1 | G2 | G2 |
| TCGA-DH-A66B | G1 | G1 | G1 |
| TCGA-14-1402 | G1 | G2 | G2 |
| TCGA-S9-A6WP | G1 | G1 | G1 |
| TCGA-HW-8321 | G1 | G1 | G1 |
| TCGA-DU-7302 | G1 | G1 | G1 |
| TCGA-FG-5963 | G1 | G1 | G2 |
| TCGA-FG-8191 | G1 | G1 | G2 |
| TCGA-KT-A7W1 | G1 | G1 | G2 |
| TCGA-QH-A870 | G1 | G1 | G1 |
| TCGA-DH-5144 | G1 | G1 | G1 |
| TCGA-HT-7483 | G1 | G1 | G1 |
| TCGA-DU-A5TY | G1 | G1 | G2 |
| TCGA-HT-A74O | G1 | G1 | G2 |
| TCGA-TQ-A7RO | G1 | G1 | G1 |
| TCGA-CS-5395 | G1 | G1 | G2 |
| TCGA-HT-7680 | G1 | G1 | G2 |
| TCGA-DB-A4XA | G1 | G1 | G1 |
| TCGA-TM-A84R | G1 | G1 | G1 |
| TCGA-DU-5853 | G1 | G1 | G1 |
| TCGA-TM-A7CF | G1 | G1 | G1 |
| TCGA-TM-A84T | G1 | G1 | G1 |
| TCGA-HT-A616 | G1 | G1 | G1 |
| TCGA-DU-5852 | G1 | G1 | G2 |
| TCGA-DU-A7TD | G1 | G1 | G2 |
| TCGA-HT-8558 | G1 | G1 | G1 |
| TCGA-FG-7637 | G1 | G1 | G1 |
| TCGA-DU-7019 | G1 | G1 | G1 |
| TCGA-HT-7676 | G1 | G1 | G1 |
| TCGA-HT-7472 | G1 | G1 | G1 |
| TCGA-IK-8125 | G1 | G1 | G1 |
| TCGA-CS-6668 | G1 | G1 | G1 |
| TCGA-P5-A5F1 | G1 | G1 | G2 |
| TCGA-DU-5871 | G1 | G1 | G1 |
| TCGA-HT-7877 | G1 | G1 | G1 |
| TCGA-06-1804 | G1 | G1 | G2 |
| TCGA-TM-A84C | G1 | G1 | G2 |
| TCGA-P5-A5EV | G1 | G1 | G2 |
| TCGA-HW-8319 | G1 | G1 | G1 |
| TCGA-HT-7479 | G1 | G1 | G1 |
| TCGA-DU-7304 | G1 | G1 | G1 |
| TCGA-R8-A73M | G1 | G1 | G1 |
| TCGA-DB-A64L | G1 | G1 | G1 |
| TCGA-32-5222 | G1 | G2 | G1 |
| TCGA-HW-A5KJ | G1 | G1 | G1 |
| TCGA-DU-6405 | G1 | G1 | G2 |
| TCGA-FG-A4MY | G1 | G1 | G1 |
| TCGA-E1-A7Z3 | G1 | G1 | G2 |
| TCGA-HT-A74L | G1 | G1 | G1 |
| TCGA-QH-A65S | G1 | G1 | G2 |
| TCGA-QH-A6X4 | G1 | G1 | G1 |
| TCGA-12-5295 | G1 | G2 | G2 |
| TCGA-DU-A7TC | G1 | G1 | G1 |
| TCGA-DU-A7TG | G1 | G1 | G1 |
| TCGA-76-4927 | G1 | G2 | G2 |
| TCGA-CS-4938 | G1 | G1 | G1 |
| TCGA-HT-A5R5 | G1 | G1 | G2 |
| TCGA-DU-5874 | G1 | G1 | G1 |
| TCGA-VM-A8CH | G1 | G1 | G1 |
| TCGA-QH-A65X | G1 | G1 | G1 |
| TCGA-CS-4941 | G1 | G1 | G2 |
| TCGA-TM-A7C3 | G1 | G1 | G2 |
| TCGA-HT-7482 | G1 | G1 | G1 |
| TCGA-FG-A4MW | G1 | G1 | G2 |
| TCGA-HT-7874 | G1 | G1 | G1 |
| TCGA-DU-6407 | G1 | G1 | G2 |
| TCGA-HT-7873 | G1 | G1 | G1 |
| TCGA-CS-4944 | G1 | G1 | G2 |
| TCGA-E1-A7Z6 | G1 | G1 | G1 |
| TCGA-HT-8563 | G1 | G1 | G2 |
| TCGA-FG-8186 | G1 | G1 | G1 |
| TCGA-HT-7474 | G1 | G1 | G1 |
| TCGA-FG-A710 | G1 | G1 | G1 |
| TCGA-TQ-A7RI | G1 | G1 | G1 |
| TCGA-DB-5274 | G1 | G1 | G1 |
| TCGA-S9-A6U6 | G1 | G1 | G1 |
| TCGA-26-1442 | G1 | G2 | G2 |
| TCGA-FG-A4MX | G1 | G1 | G2 |
| TCGA-HT-7688 | G1 | G1 | G1 |
| TCGA-P5-A5F4 | G1 | G1 | G1 |
| TCGA-WY-A85C | G1 | G1 | G1 |
| TCGA-FG-A4MT | G1 | G1 | G1 |
| TCGA-FG-7641 | G1 | G1 | G1 |
| TCGA-DU-A7T8 | G1 | G1 | G2 |
| TCGA-FG-A70Y | G1 | G1 | G1 |
| TCGA-DH-A7UT | G1 | G1 | G2 |
| TCGA-FG-A87N | G1 | G1 | G2 |
| TCGA-DB-A75K | G1 | G1 | G1 |
| TCGA-DH-A7US | G1 | G1 | G1 |
| TCGA-HT-7882 | G1 | G1 | G2 |
| TCGA-DB-A4XG | G1 | G1 | G1 |
| TCGA-DU-6393 | G1 | G1 | G1 |
| TCGA-RY-A83Y | G1 | G1 | G1 |
| TCGA-28-5208 | G1 | G2 | G2 |
| TCGA-DU-A7TJ | G1 | G1 | G2 |
| TCGA-HT-7875 | G1 | G1 | G1 |
| TCGA-DH-A669 | G1 | G1 | G1 |
| TCGA-DU-7298 | G1 | G1 | G1 |
| TCGA-QH-A6X5 | G1 | G1 | G1 |
| TCGA-P5-A733 | G1 | G1 | G1 |
| TCGA-F6-A8O3 | G1 | G1 | G1 |
| TCGA-06-0171 | G1 | G2 | G2 |
| TCGA-TM-A84S | G1 | G1 | G2 |
| TCGA-HT-7601 | G1 | G1 | G2 |
| TCGA-HT-7857 | G1 | G1 | G2 |
| TCGA-E1-A7YJ | G1 | G1 | G2 |
| TCGA-DH-A7UR | G1 | G1 | G1 |
| TCGA-E1-A7YO | G1 | G1 | G1 |
| TCGA-E1-5304 | G1 | G1 | G1 |
| TCGA-76-4926 | G1 | G2 | G2 |
| TCGA-E1-A7YV | G1 | G1 | G2 |
| TCGA-TQ-A8XE | G1 | G1 | G1 |
| TCGA-S9-A7IY | G1 | G1 | G1 |
| TCGA-DU-6403 | G1 | G1 | G2 |
| TCGA-RY-A83Z | G1 | G1 | G2 |
| TCGA-DH-5143 | G1 | G1 | G1 |
| TCGA-HT-8015 | G1 | G1 | G2 |
| TCGA-VM-A8CB | G1 | G1 | G1 |
| TCGA-FG-7636 | G1 | G1 | G1 |
| TCGA-DU-5872 | G1 | G1 | G2 |
| TCGA-FG-8188 | G1 | G1 | G1 |
| TCGA-TM-A84M | G1 | G1 | G1 |
| TCGA-FG-A6J1 | G1 | G1 | G1 |
| TCGA-P5-A781 | G1 | G1 | G1 |
| TCGA-P5-A737 | G1 | G1 | G1 |
| TCGA-DU-5847 | G1 | G1 | G2 |
| TCGA-RY-A843 | G1 | G1 | G1 |
| TCGA-06-5408 | G1 | G2 | G2 |
| TCGA-HT-7677 | G1 | G1 | G1 |
| TCGA-P5-A731 | G1 | G1 | G2 |
| TCGA-S9-A7R4 | G1 | G1 | G1 |
| TCGA-DB-A64W | G1 | G1 | G1 |
| TCGA-QH-A86X | G1 | G1 | G1 |
| TCGA-S9-A6UA | G1 | G1 | G2 |
| TCGA-HT-A615 | G1 | G1 | G1 |
| TCGA-E1-A7Z2 | G1 | G1 | G2 |
| TCGA-06-0190 | G1 | G2 | G2 |
| TCGA-P5-A72X | G1 | G1 | G2 |
| TCGA-HT-7681 | G1 | G1 | G1 |
| TCGA-HT-8114 | G1 | G1 | G1 |
| TCGA-S9-A6TS | G1 | G1 | G2 |
| TCGA-HW-7491 | G1 | G1 | G1 |
| TCGA-P5-A780 | G1 | G1 | G1 |
| TCGA-HT-7478 | G1 | G1 | G2 |
| TCGA-HT-7475 | G1 | G1 | G1 |
| TCGA-HW-7495 | G1 | G1 | G1 |
| TCGA-HT-7691 | G1 | G1 | G1 |
| TCGA-26-5133 | G1 | G2 | G2 |
| TCGA-CS-5397 | G1 | G1 | G2 |
| TCGA-TM-A84I | G1 | G1 | G1 |
| TCGA-CS-4942 | G1 | G1 | G1 |
| TCGA-R8-A6MK | G1 | G1 | G1 |
| TCGA-DB-A64S | G1 | G1 | G2 |
| TCGA-WY-A85D | G1 | G1 | G2 |
| TCGA-DU-6408 | G1 | G1 | G1 |
| TCGA-DU-A76L | G1 | G1 | G2 |
| TCGA-VM-A8C8 | G1 | G1 | G1 |
| TCGA-DU-6399 | G1 | G1 | G1 |
| TCGA-HT-7609 | G1 | G1 | G1 |
| TCGA-HT-A5RA | G1 | G1 | G2 |
| TCGA-DU-A7T6 | G1 | G1 | G1 |
| TCGA-DU-7301 | G1 | G1 | G1 |
| TCGA-FG-8185 | G1 | G1 | G1 |
| TCGA-QH-A65V | G1 | G1 | G1 |
| TCGA-TM-A7C5 | G1 | G1 | G1 |
| TCGA-S9-A7R7 | G1 | G1 | G2 |
| TCGA-DB-A4XF | G1 | G1 | G1 |
| TCGA-S9-A7R2 | G1 | G1 | G2 |
| TCGA-QH-A6X3 | G1 | G1 | G1 |
| TCGA-E1-A7YN | G1 | G1 | G2 |
| TCGA-FG-5965 | G1 | G1 | G1 |
| TCGA-S9-A6U1 | G1 | G1 | G1 |
| TCGA-TQ-A7RG | G1 | G1 | G1 |
| TCGA-19-0957 | G1 | G1 | G2 |
| TCGA-DU-7014 | G1 | G1 | G1 |
| TCGA-FG-8187 | G1 | G1 | G1 |
| TCGA-HT-7477 | G1 | G1 | G2 |
| TCGA-F6-A8O4 | G1 | G1 | G1 |
| TCGA-FG-6689 | G1 | G1 | G1 |
| TCGA-06-5411 | G1 | G2 | G2 |
| TCGA-R8-A6MO | G1 | G1 | G1 |
| TCGA-P5-A5EY | G1 | G1 | G2 |
| TCGA-DU-6406 | G1 | G1 | G2 |
| TCGA-DB-A4XE | G1 | G1 | G1 |
| TCGA-HW-A5KL | G1 | G1 | G1 |
| TCGA-12-5299 | G1 | G2 | G2 |
| TCGA-S9-A6WL | G1 | G1 | G1 |
| TCGA-DU-5849 | G1 | G1 | G1 |
| TCGA-HT-A61A | G1 | G1 | G1 |
| TCGA-28-5204 | G1 | G2 | G2 |
| TCGA-E1-5307 | G1 | G1 | G1 |
| TCGA-FG-8189 | G1 | G1 | G1 |
| TCGA-HW-A5KK | G1 | G1 | G2 |
| TCGA-E1-A7YS | G1 | G1 | G1 |
| TCGA-RY-A845 | G1 | G1 | G1 |
| TCGA-HT-A5RC | G1 | G1 | G2 |
| TCGA-HT-7860 | G1 | G1 | G2 |
| TCGA-RY-A83X | G1 | G1 | G1 |
| TCGA-DU-7294 | G1 | G1 | G1 |
| TCGA-TM-A7C4 | G1 | G1 | G1 |
| TCGA-TQ-A7RR | G1 | G1 | G2 |
| TCGA-06-0221 | G1 | G1 | G2 |
| TCGA-HW-7490 | G1 | G1 | G1 |
| TCGA-DB-A64Q | G1 | G1 | G1 |
| TCGA-76-4929 | G1 | G2 | G2 |
| TCGA-DU-7012 | G1 | G1 | G2 |
| TCGA-P5-A5F0 | G1 | G1 | G1 |
| TCGA-HT-7602 | G1 | G1 | G1 |
| TCGA-DB-A75O | G1 | G1 | G2 |
| TCGA-TQ-A7RJ | G1 | G1 | G1 |
| TCGA-S9-A6WM | G1 | G1 | G2 |
| TCGA-DU-6542 | G1 | G1 | G1 |
| TCGA-HT-A617 | G1 | G1 | G2 |
| TCGA-FG-7643 | G1 | G1 | G2 |
| TCGA-HT-7686 | G1 | G1 | G2 |
| TCGA-E1-5303 | G1 | G1 | G2 |
| TCGA-DU-A5TP | G1 | G1 | G2 |
| TCGA-S9-A7QY | G1 | G1 | G1 |
| TCGA-DU-7299 | G1 | G1 | G1 |
| TCGA-FG-A711 | G1 | G1 | G1 |
| TCGA-HT-A74H | G1 | G1 | G2 |
| TCGA-TQ-A7RQ | G1 | G1 | G1 |
| TCGA-QH-A6CS | G1 | G1 | G2 |
| TCGA-VM-A8C9 | G1 | G1 | G1 |
| TCGA-HT-A4DS | G1 | G1 | G2 |
| TCGA-DU-7009 | G1 | G1 | G1 |
| TCGA-TQ-A7RN | G1 | G1 | G1 |
| TCGA-S9-A6TU | G1 | G1 | G1 |
| TCGA-P5-A730 | G1 | G1 | G1 |
| TCGA-E1-A7YU | G1 | G1 | G1 |
| TCGA-E1-A7YY | G1 | G1 | G1 |
| TCGA-HT-8107 | G1 | G1 | G1 |
| TCGA-HT-8564 | G1 | G1 | G2 |
| TCGA-HT-A61B | G1 | G1 | G2 |
| TCGA-QH-A6X9 | G1 | G1 | G1 |
| TCGA-DB-A64V | G1 | G1 | G1 |
| TCGA-HT-7689 | G1 | G1 | G1 |
| TCGA-HT-7468 | G1 | G1 | G1 |
| TCGA-76-4932 | G1 | G2 | G2 |
| TCGA-E1-A7YK | G1 | G1 | G2 |
| TCGA-06-5412 | G1 | G2 | G2 |
| TCGA-S9-A7J0 | G1 | G1 | G1 |
| TCGA-HW-8322 | G1 | G1 | G1 |
| TCGA-DH-A66G | G1 | G1 | G1 |
| TCGA-06-5418 | G1 | G2 | G2 |
| TCGA-DU-5870 | G1 | G1 | G1 |
| TCGA-HT-8109 | G1 | G1 | G1 |
| TCGA-DU-8166 | G1 | G1 | G1 |
| TCGA-FG-A60K | G1 | G1 | G1 |
| TCGA-HT-7603 | G1 | G1 | G1 |
| TCGA-HT-8104 | G1 | G1 | G2 |
| TCGA-26-5132 | G1 | G2 | G2 |
| TCGA-HT-7480 | G1 | G1 | G1 |
| TCGA-S9-A7J1 | G1 | G1 | G1 |
| TCGA-DB-A4XB | G1 | G1 | G1 |
| TCGA-S9-A6TV | G1 | G1 | G2 |
| TCGA-E1-A7YD | G1 | G1 | G2 |
| TCGA-DU-8168 | G1 | G1 | G1 |
| TCGA-HT-8012 | G1 | G1 | G1 |
| TCGA-HT-7884 | G1 | G1 | G1 |
| TCGA-DB-A75L | G1 | G1 | G1 |
| TCGA-DU-7290 | G1 | G1 | G2 |
| TCGA-DU-6392 | G1 | G1 | G2 |
| TCGA-CS-5394 | G1 | G1 | G1 |
| TCGA-DB-5280 | G1 | G1 | G1 |
| TCGA-DU-7007 | G1 | G1 | G2 |
| TCGA-HT-7687 | G1 | G1 | G1 |
| TCGA-QH-A6CZ | G1 | G1 | G1 |
| TCGA-VV-A86M | G1 | G1 | G1 |
| TCGA-FN-7833 | G1 | G1 | G2 |
| TCGA-06-5859 | G1 | G2 | G2 |
| TCGA-S9-A7J2 | G1 | G1 | G1 |
| TCGA-DH-5142 | G1 | G1 | G1 |
| TCGA-FG-5964 | G1 | G1 | G1 |
| TCGA-HT-7467 | G1 | G1 | G1 |
| TCGA-28-5216 | G1 | G2 | G2 |
| TCGA-FG-A713 | G1 | G1 | G1 |
| TCGA-HT-7608 | G1 | G1 | G1 |
| TCGA-HW-7487 | G1 | G1 | G1 |
| TCGA-S9-A6U5 | G1 | G1 | G1 |
| TCGA-DU-A7TA | G1 | G1 | G2 |
| TCGA-S9-A6U8 | G1 | G1 | G1 |
| TCGA-S9-A7R8 | G1 | G1 | G1 |
| TCGA-HT-8108 | G1 | G1 | G1 |
| TCGA-DH-A66D | G1 | G1 | G1 |
| TCGA-S9-A7IZ | G1 | G1 | G2 |
| TCGA-26-5135 | G1 | G2 | G2 |
| TCGA-QH-A6XC | G1 | G1 | G2 |
| TCGA-QH-A6CU | G1 | G1 | G2 |
| TCGA-RY-A840 | G1 | G1 | G1 |
| TCGA-DU-7300 | G1 | G1 | G1 |
| TCGA-WY-A859 | G1 | G1 | G1 |
| TCGA-06-5416 | G1 | G2 | G2 |
| TCGA-HT-A614 | G1 | G1 | G1 |
| TCGA-P5-A5F2 | G1 | G1 | G1 |
| TCGA-DB-5279 | G1 | G1 | G1 |
| TCGA-HT-A61C | G1 | G1 | G2 |
| TCGA-S9-A7J3 | G1 | G1 | G1 |
| TCGA-S9-A6TX | G1 | G1 | G1 |
| TCGA-HT-7854 | G1 | G1 | G2 |
| TCGA-26-5134 | G1 | G2 | G2 |
| TCGA-06-0152 | G1 | G2 | G2 |
| TCGA-S9-A7QW | G1 | G1 | G1 |
| TCGA-FG-6691 | G1 | G1 | G1 |
| TCGA-DU-A7TB | G1 | G1 | G1 |
| TCGA-R8-A6ML | G1 | G1 | G1 |
| TCGA-DB-5273 | G1 | G1 | G1 |
| TCGA-VM-A8CD | G1 | G1 | G2 |
| TCGA-DH-5140 | G1 | G1 | G2 |
| TCGA-VM-A8CE | G1 | G1 | G1 |
| TCGA-DU-7018 | G1 | G1 | G1 |
| TCGA-HT-A619 | G1 | G1 | G1 |
| TCGA-P5-A735 | G1 | G1 | G1 |
| TCGA-DB-A64O | G1 | G1 | G2 |
| TCGA-FG-A60J | G1 | G1 | G1 |
| TCGA-HW-7486 | G1 | G1 | G1 |
| TCGA-DU-A5TT | G1 | G1 | G2 |
| TCGA-E1-A7YQ | G1 | G1 | G2 |
| TCGA-TM-A84F | G1 | G1 | G2 |
| TCGA-HT-7485 | G1 | G1 | G1 |
| TCGA-DU-7006 | G1 | G1 | G2 |
| TCGA-S9-A6U9 | G1 | G1 | G1 |
| TCGA-HT-7473 | G1 | G1 | G1 |
| TCGA-HT-7606 | G1 | G1 | G1 |
| TCGA-S9-A7IQ | G1 | G1 | G1 |
| TCGA-E1-A7YH | G1 | G1 | G1 |
| TCGA-FG-A60L | G1 | G1 | G2 |
| TCGA-E1-5302 | G1 | G1 | G1 |
| TCGA-HW-8320 | G1 | G1 | G1 |
| TCGA-DU-7010 | G1 | G1 | G2 |
| TCGA-P5-A5EZ | G1 | G1 | G2 |
| TCGA-HT-A74J | G1 | G1 | G1 |
| TCGA-76-4928 | G1 | G2 | G2 |
| TCGA-06-5413 | G1 | G2 | G2 |
| TCGA-P5-A5EW | G1 | G1 | G1 |
| TCGA-DU-8161 | G1 | G1 | G2 |
| TCGA-QH-A65R | G1 | G1 | G1 |
| TCGA-VM-A8CF | G1 | G1 | G2 |
| TCGA-DB-A75M | G1 | G1 | G1 |
| TCGA-19-5960 | G1 | G2 | G2 |
| TCGA-S9-A89V | G1 | G1 | G2 |
| TCGA-DB-A64P | G1 | G1 | G1 |
| TCGA-DU-7008 | G1 | G1 | G2 |
| TCGA-HT-7470 | G1 | G1 | G1 |
| TCGA-DU-7011 | G1 | G1 | G1 |
| TCGA-06-0210 | G1 | G2 | G2 |
| TCGA-HW-A5KM | G1 | G1 | G2 |
| TCGA-TQ-A7RP | G1 | G1 | G2 |
| TCGA-HT-8110 | G1 | G1 | G2 |
| TCGA-HT-8013 | G1 | G1 | G1 |
| TCGA-28-5209 | G1 | G2 | G2 |
| TCGA-E1-5311 | G1 | G1 | G1 |
| TCGA-41-5651 | G1 | G2 | G2 |
| TCGA-DU-A6S6 | G1 | G1 | G1 |
| TCGA-HT-7694 | G1 | G1 | G1 |
| TCGA-HT-7607 | G1 | G1 | G1 |
| TCGA-S9-A6WG | G1 | G1 | G2 |
| TCGA-CS-4943 | G1 | G1 | G1 |
| TCGA-DH-A7UV | G1 | G1 | G1 |
| TCGA-DU-5854 | G1 | G1 | G2 |
| TCGA-DU-8158 | G1 | G1 | G2 |
| TCGA-QH-A6X8 | G1 | G1 | G1 |
| TCGA-FG-6690 | G1 | G1 | G1 |
| TCGA-FG-7638 | G1 | G1 | G1 |
| TCGA-TQ-A7RV | G1 | G1 | G1 |
| TCGA-DU-A6S8 | G1 | G1 | G2 |
| TCGA-DB-A75P | G1 | G1 | G2 |
| TCGA-W9-A837 | G1 | G1 | G1 |
| TCGA-DB-A64R | G1 | G1 | G1 |
| TCGA-DH-5141 | G1 | G1 | G1 |
| TCGA-14-0781 | G1 | G2 | G2 |
| TCGA-HT-8113 | G1 | G1 | G1 |
| TCGA-DU-8162 | G1 | G1 | G2 |
| TCGA-S9-A6WQ | G1 | G1 | G1 |
| TCGA-QH-A6CY | G1 | G1 | G1 |
| TCGA-S9-A7IS | G1 | G1 | G1 |
| TCGA-HT-8019 | G1 | G1 | G1 |
| TCGA-E1-A7YI | G1 | G1 | G1 |
| TCGA-WY-A85A | G1 | G1 | G1 |
| TCGA-26-5136 | G1 | G2 | G2 |
| TCGA-VW-A7QS | G1 | G1 | G1 |
| TCGA-TQ-A7RK | G1 | G1 | G1 |
| TCGA-TM-A84O | G1 | G1 | G1 |
| TCGA-HT-7471 | G1 | G1 | G1 |
| TCGA-S9-A6WO | G1 | G1 | G1 |
| TCGA-DU-6410 | G1 | G1 | G1 |
| TCGA-FG-6692 | G1 | G1 | G2 |
| TCGA-DU-A76K | G1 | G1 | G2 |
| TCGA-WY-A85B | G1 | G1 | G1 |
| TCGA-28-5207 | G1 | G2 | G2 |
| TCGA-DU-A5TR | G1 | G1 | G2 |
| TCGA-DU-5855 | G1 | G1 | G1 |
| TCGA-DU-8167 | G1 | G1 | G1 |
| TCGA-HT-7620 | G1 | G1 | G1 |
| TCGA-15-1444 | G1 | G2 | G2 |
| TCGA-DB-A4XC | G1 | G1 | G1 |
| TCGA-DU-A5TU | G1 | G1 | G2 |
| TCGA-QH-A6XA | G1 | G1 | G1 |
| TCGA-DB-5278 | G1 | G1 | G1 |
| TCGA-E1-A7Z4 | G1 | G1 | G1 |
| TCGA-HT-7469 | G1 | G1 | G2 |
| TCGA-EZ-7264 | G1 | G1 | G1 |
| TCGA-WY-A858 | G1 | G1 | G2 |
| TCGA-28-5220 | G1 | G2 | G2 |
| TCGA-E1-5319 | G1 | G1 | G1 |
| TCGA-14-0736 | G2 | G2 | G2 |
| TCGA-CS-6186 | G1 | G1 | G2 |
| TCGA-WY-A85E | G1 | G1 | G1 |
| TCGA-19-4065 | G1 | G2 | G2 |
| TCGA-IK-7675 | G1 | G1 | G1 |
| TCGA-CS-6667 | G1 | G1 | G1 |
| TCGA-HT-7690 | G1 | G1 | G2 |
| TCGA-DU-7292 | G1 | G1 | G2 |
| TCGA-DU-6400 | G1 | G1 | G1 |
| TCGA-S9-A7QX | G1 | G1 | G1 |
| TCGA-S9-A6U0 | G1 | G1 | G2 |
| TCGA-HT-7856 | G1 | G1 | G1 |
| TCGA-DB-A4XD | G1 | G1 | G1 |
| TCGA-P5-A72W | G1 | G1 | G1 |
| TCGA-DB-5281 | G1 | G1 | G1 |
| TCGA-TM-A84G | G1 | G1 | G1 |
| TCGA-P5-A77X | G1 | G1 | G1 |
| TCGA-S9-A89Z | G1 | G1 | G1 |
| TCGA-P5-A72U | G1 | G1 | G2 |
| TCGA-HT-7610 | G1 | G1 | G1 |
| TCGA-06-0125 | G1 | G2 | G2 |
| TCGA-DH-A66F | G1 | G1 | G1 |
| TCGA-E1-A7YE | G1 | G1 | G2 |
| TCGA-QH-A6CX | G1 | G1 | G2 |
| TCGA-TM-A84H | G1 | G1 | G1 |
| TCGA-DU-A76R | G1 | G1 | G1 |
| TCGA-06-5414 | G1 | G2 | G2 |
