# Supplementary material for: Deep learning algorithm reveals two prognostic subtypes in patients with gliomas
Source: BMC Bioinformatics. 2022 Oct 11;23:417. doi: 10.1186/s12859-022-04970-x (PMC9552440; doi:10.1186/s12859-022-04970-x)

**Supplementary Files**

**Additional File 6**

**Figure S3**. Kaplan-Meier survival curves of three approaches. (a) Autoencoder-based approach. (b) iCluster. (c) PCA.


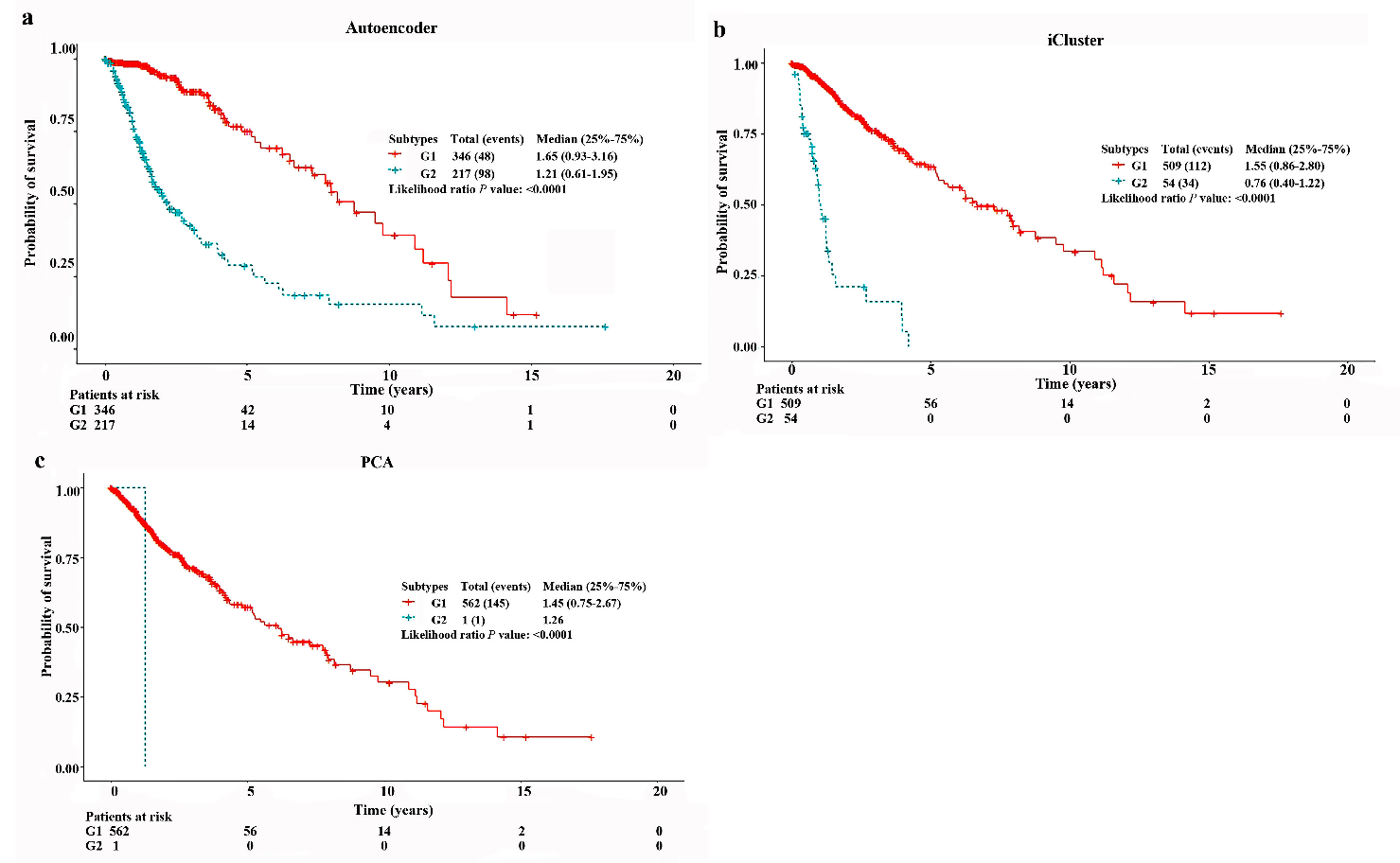

Supplement: Supplementary file 6 — Additional file 6: Figure S3. Kaplan-Meier survival curves of three approaches. (a) Autoencoder-based approach. (b) iCluster. (c) PCA. [file 12859_2022_4970_MOESM6_ESM.docx]
