# Supplementary material for: Deep learning algorithm reveals two prognostic subtypes in patients with gliomas
Source: BMC Bioinformatics. 2022 Oct 11;23:417. doi: 10.1186/s12859-022-04970-x (PMC9552440; doi:10.1186/s12859-022-04970-x)
Supplement: Supplementary file 7 — Additional file 7: Table S4. Cross validation-based performance of the SVM model when using alternative approaches. [file 12859_2022_4970_MOESM7_ESM.docx]

**Supplementary Files**

**Additional File 7**

**Table S4**. Cross validation-based performance of the SVM model when using alternative approaches

| Alternative approaches | C-index,  mean (SD) | Brier score,  mean (SD) | Log-rank *p* value, geo.mean |
| --- | --- | --- | --- |
| Autoencoder | 0.92 (0.02) | 0.16 (0.02) | 4.68E-12 |
| iCluster | 0.90 (0.02) | 0.15 (0.01) | 2.89E-11 |

SVM, support vector machine; geo.mean, geometric mean
