# Supplementary material for: Deep learning algorithm reveals two prognostic subtypes in patients with gliomas
Source: BMC Bioinformatics. 2022 Oct 11;23:417. doi: 10.1186/s12859-022-04970-x (PMC9552440; doi:10.1186/s12859-022-04970-x)
Supplement: Supplementary file 10 — Additional file 10: Table S7. Clinical information of CGGA DNAm dataset. [file 12859_2022_4970_MOESM10_ESM.docx]

**Supplementary Files**

**Additional File 10**

**Table S7**. Clinical information of CGGA DNAm dataset

| Clinical information | G1 (16) | G2 (124) | *p* value |
| --- | --- | --- | --- |
| Age, mean (SD), median (25%–75%), years | 38 (34-45) | 39 (32-50) | 0.67 |
| Men, N (%) | 9 (56.25) | 71 (57.26) | 0.94 |
| Tumor grades, N (%) |  |  |  |
| 2 | 12 (75.00) | 42 (33.87) | 0.01 |
| 3 | 3 (18.75) | 41 (33.06) |  |
| 4 | 1 (6.25) | 41 (33.06) |  |
| Tumor types, N (%) |  |  |  |
| GBM | 1 (6.25) | 41 (33.06) | 0.06 |
| Others | 15 (93.75) | 83 (66.94) |  |
| Follow-up time, median (25%–75%), years | 4.35 (2.40-11.41) | 3.06 (0.98-9.93) | 0.12 |
| Dead, N (%) | 6 (37.50) | 84 (67.74) | 0.02 |
| *IDH* mutant | 8 (50.00) | 66 (53.23) | 0.81 |

The clinical information is described as frequencies with percentages for categorical variables and medians with interquartile ranges (IQRs) for continuous variables. The statistical differences between two subtypes were determined using Wilcoxon rank-sum tests for continuous variables and χ2 tests for categorical variables
