# Supplementary material for: Deep learning algorithm reveals two prognostic subtypes in patients with gliomas
Source: BMC Bioinformatics. 2022 Oct 11;23:417. doi: 10.1186/s12859-022-04970-x (PMC9552440; doi:10.1186/s12859-022-04970-x)
Supplement: Supplementary file 11 — Additional file 11: Table S8. Performance of the model based on clinical features. [file 12859_2022_4970_MOESM11_ESM.docx]

**Supplementary Files**

**Additional File 11**

**Table S8**. Performance of the model based on clinical features

| Dataset | C-index | Brier score | Log-rank *p* value |
| --- | --- | --- | --- |
| TCGA (G2 vs G1) | 0.91 | 0.17 | <0.0001 |
| TCGA (GBM vs LGG) | 0.90 | 0.15 | <0.0001 |
| TCGA (Combind) | 0.86 | 0.14 | <0.0001 |

Combind model included age, gender, tumor grade, tumor types and autoencoder subtypes
