# Supplementary material for: Deep learning algorithm reveals two prognostic subtypes in patients with gliomas
Source: BMC Bioinformatics. 2022 Oct 11;23:417. doi: 10.1186/s12859-022-04970-x (PMC9552440; doi:10.1186/s12859-022-04970-x)
Supplement: Supplementary file 12 — Additional file 12: Figure S4. mRNA levels of DNA methylation driven genes. [file 12859_2022_4970_MOESM12_ESM.docx]

**Supplementary Files**

**Additional File 12**

**Figure S4**. mRNA levels of DNA methylation driven genes


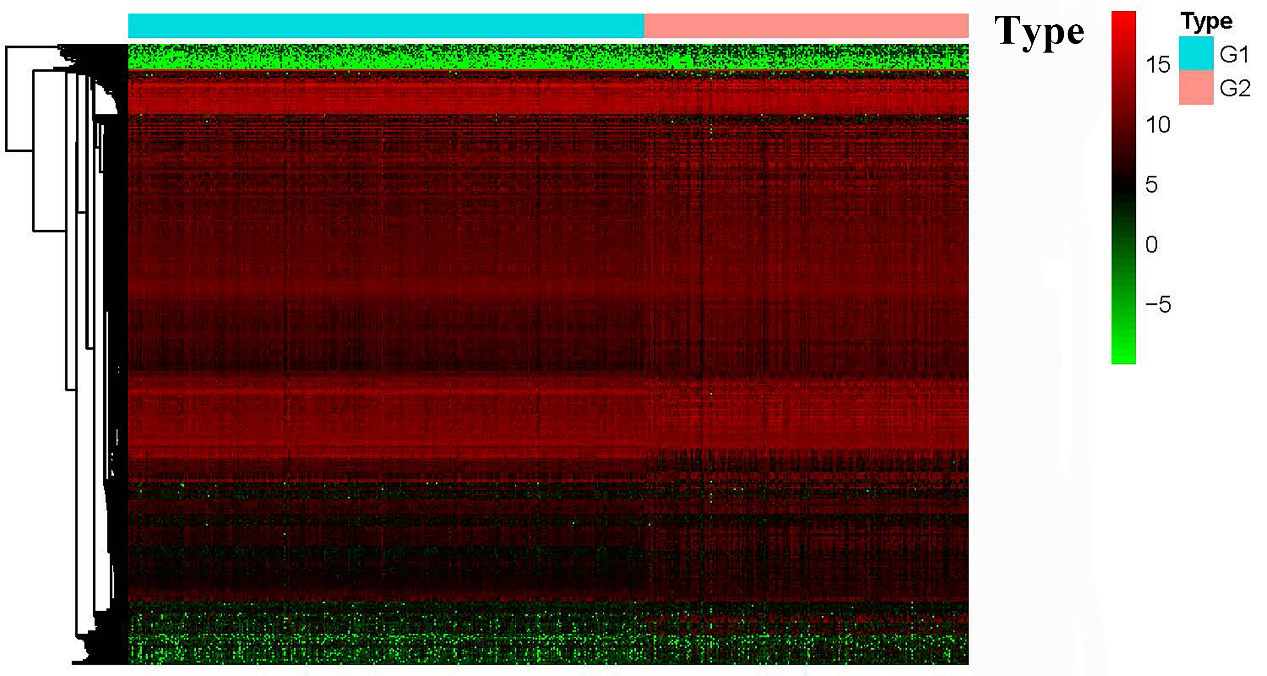
Figure S4. mRNA levels of DNA methylation driven genes
