# Supplementary material for: Deep learning algorithm reveals two prognostic subtypes in patients with gliomas
Source: BMC Bioinformatics. 2022 Oct 11;23:417. doi: 10.1186/s12859-022-04970-x (PMC9552440; doi:10.1186/s12859-022-04970-x)

**Supplementary Files**

**Additional File 13**

**Figure S5**. Methylation levels of DNA methylation driven genes


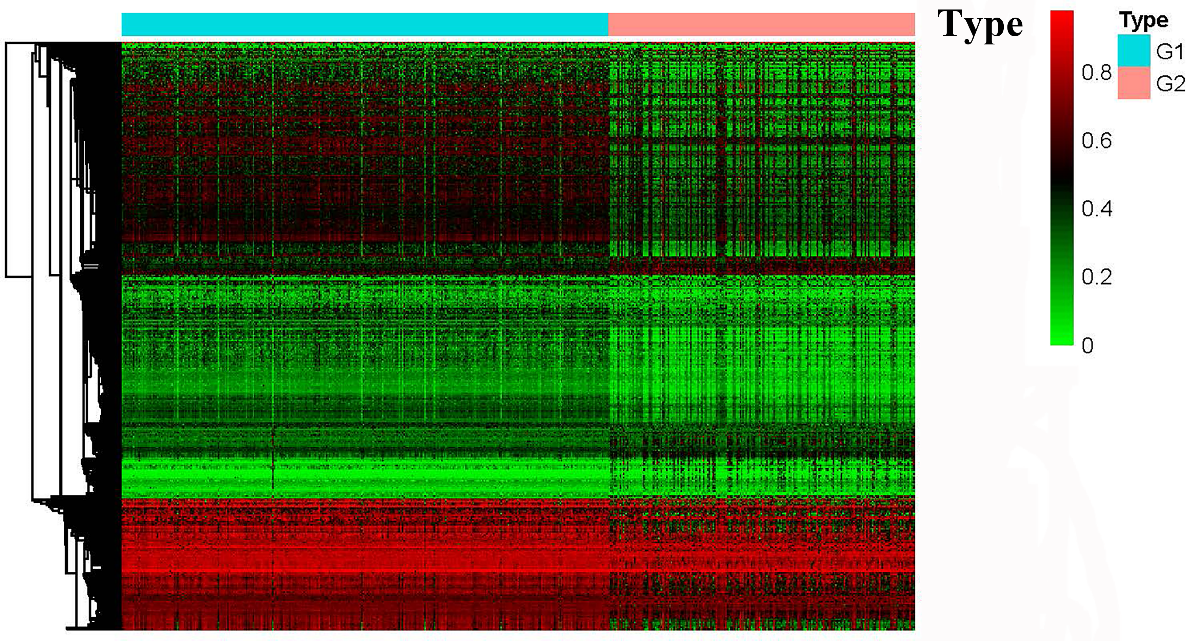

Supplement: Supplementary file 13 — Additional file 13: Figure S5. Methylation levels of DNA methylation driven genes. [file 12859_2022_4970_MOESM13_ESM.docx]
