# Supplementary material for: Deep learning algorithm reveals two prognostic subtypes in patients with gliomas
Source: BMC Bioinformatics. 2022 Oct 11;23:417. doi: 10.1186/s12859-022-04970-x (PMC9552440; doi:10.1186/s12859-022-04970-x)
Supplement: Supplementary file 14 — Additional file 14: Table S9. KEGG pathway analysis of DNAm-driven genes. [file 12859_2022_4970_MOESM14_ESM.docx]

**Supplementary Files**

**Additional File 14**

**Table S9**. KEGG pathway analysis of DNAm-driven genes

| Pathway | Source | members_input_overlap | Size | *p* value |
| --- | --- | --- | --- | --- |
| Herpes simplex virus 1 infection | KEGG | ZNF714; ZNF717; ZNF233; ZFP69;  ZNF257; ZNF208; ZNF135 | 498 | 0.002 |
| Axon guidance | KEGG | RGMA; NFATC4; CXCR4; SSH3;  MYL12A; FES; RHOD; SEMA3E | 182 | 0.003 |
| Salmonella infection | KEGG | MYD88; CASP4; MYL12A; ANXA2;  S100A10; CASP1; BIRC3; DYNLT3; NCKAP1L | 249 | 0.006 |
| Glutathione metabolism | KEGG | GSTM1; GSTM5; MGST2; OPLAH | 57 | 0.007 |
