# Supplementary material for: Deep learning algorithm reveals two prognostic subtypes in patients with gliomas
Source: BMC Bioinformatics. 2022 Oct 11;23:417. doi: 10.1186/s12859-022-04970-x (PMC9552440; doi:10.1186/s12859-022-04970-x)
Supplement: Supplementary file 15 — Additional file 15: Supplementary methods. Description of normalization methods used data normalization. [file 12859_2022_4970_MOESM15_ESM.docx]

**Supplementary Files**

**Additional File 15**

**Supplementary methods**. Description of normalization methods used data normalization

We applied 2-step normalization on both training and validation datasets. First, we used the median scale (MAD) normalization on both the training and validation datasets. Second, we applied the robust scale normalization on the training dataset, and scaled the validation dataset using the means and standard deviations of the training dataset.

**Median scale normalization**: The median absolute deviation (*mad*) is a robust estimator of the variability of a univariate sample. For a given feature vector $x=(x_{1},\ldots,x_{n})$:

$$mad\left( x \right)=median(\left\{ \left| x_{i}-median(x) \right|, x_{i}\in x \right\})$$

The “median scale normalization” is defined by:

$$x_{scaled}=\left( x-median\left( x \right) \right).\frac{1}{mad(x)}$$

**Robust scale normalization**: For each feature, the mean and the standard deviation is computed using values between the first and the last quantile from the training set (in the case of an ordered array of values, values used begin after the first 25% until reaching 75% of the total values).

$$x_{whitened}= \left\{ \frac{x_{i}-{mean}_{25-75}(x_{i})}{{std}_{25-75}(x_{i})}, x_{i}\in x \right\}$$

This normalization presents the advantage to compute robust mean and standard deviation estimates without being influenced by possible outliers.
